# What 5000 babies can tell us about developing minds and how to study them

**DOI:** 10.1038/s44271-026-00477-w

**Published:** 2026-06-09

**Authors:** Brianna T. M. McMillan, Heidi A. Baumgartner, Christina Bergmann, Michael C. Frank, J. Kiley Hamlin, Dora Kampis, Melissa Kline Struhl, Eon-Suk Ko, Jessica E. Kosie, Casey Lew-Williams, Kelsey Lucca, Tobias Schuwerk, Melanie Soderstrom, Ingmar Visser, Francis L. Yuen, Martin Zettersten, Krista Byers-Heinlein

**Affiliations:** 1 Department of Psychology, Smith College, Northampton, MA USA; 2 Department of Psychology, Stanford University, Stanford, CA USA; 3 Osnabrück University of Applied Sciences, Osnabrück, Germany; 4 Max Planck Institute for Psycholinguistics, Nijmegen, Netherlands; 5 Department of Psychology, University of British Columbia, Vancouver, BC Canada; 6 Department of Psychology, University of Copenhagen, Copenhagen, Denmark; 7 Department of Brain & Cognitive Sciences, Massachusetts Institute of Technology, Cambridge, MA USA; 8 Department of English Language and Literature, Chosun University, Gwangju, South Korea; 9 School of Social and Behavioral Sciences, Arizona State University, Phoenix, AZ USA; 10 Department of Psychology, Princeton University, Princeton, NJ USA; 11 Department of Psychology, Arizona State University, Tempe, AZ USA; 12 Department of Psychology, Ludwig-Maximilians-Universität München, Munich, Germany; 13 Department of Psychology, University of Manitoba, Winnipeg, MB Canada; 14 Department of Psychology, University of Amsterdam, Amsterdam, Netherlands; 15 Department of Cognitive Science, University of California San Diego, San Diego, CA USA; 16 Department of Psychology, Concordia University, Montréal, QC Canada

**Keywords:** Psychology, Human behaviour

## Abstract

A decade of ManyBabies research, testing thousands of babies across hundreds of labs, has shown that some, but not all findings in infant research replicate well. Collectively, these projects have shown that current methods carry limitations that larger samples alone cannot resolve. Here three lessons that point toward a more reliable, inclusive developmental science are presented.

## Teaser

A decade of ManyBabies research, testing thousands of babies across hundreds of labs, has shown that some, but not all findings in infant research replicate well. Collectively, these projects have shown us that our methods carry limitations that larger samples alone cannot resolve. Here we present three lessons that point toward a more reliable, inclusive developmental science.

Research on infant cognitive development seeks to understand how the mind is structured, how it changes with experience, and what this reveals about human psychology more broadly. For much of its history, infant studies have been almost exclusively conducted in individual labs with a relatively small number of infants. The single-lab model yields remarkable insights but produces work that is often small in scale, difficult to replicate, narrow in cultural scope, and hard to generalize across populations.

The ManyBabies Consortium was founded in 2015 to tackle these challenges^1^, and has grown to involve over 800 researchers in more than 50 countries across six continents. Like the ManyLabs projects^2^, the initial goal was replication at scale. However, babies are not straightforward participants: they have short, unpredictable attention spans, limited behavioral repertoires, and, because of their reliance on caregivers, are difficult to study in controlled experimental settings. By studying thousands of babies across more than 200 labs worldwide, the challenges of infant research have come into sharper focus. In the first decade of ManyBabies research, three lessons have emerged that reshaped how we think about studying infant development: large variability across infants, labs, and contexts turned out to be the norm, not the exception (Lesson 1); larger samples sharpened rather than resolved our measurement challenges (Lesson 2); and methodological choices are cultural choices (Lesson 3).

## Lesson 1: variability is data

In a typical infant experiment, the infant sits on the caregiver’s lap in a quiet room, and a researcher presents them with stimuli, such as audio clips, images on a screen, or a researcher engaging the infant across a table. Unlike adult participants, infants cannot follow instructions or report thoughts and feelings, so researchers typically rely on observable behaviors, such as whether infants look longer at one stimulus than another, reach toward it, or turn away. These subtle, fleeting behavioral responses are the primary data of behavioral infant research.

**Fig. 1:**
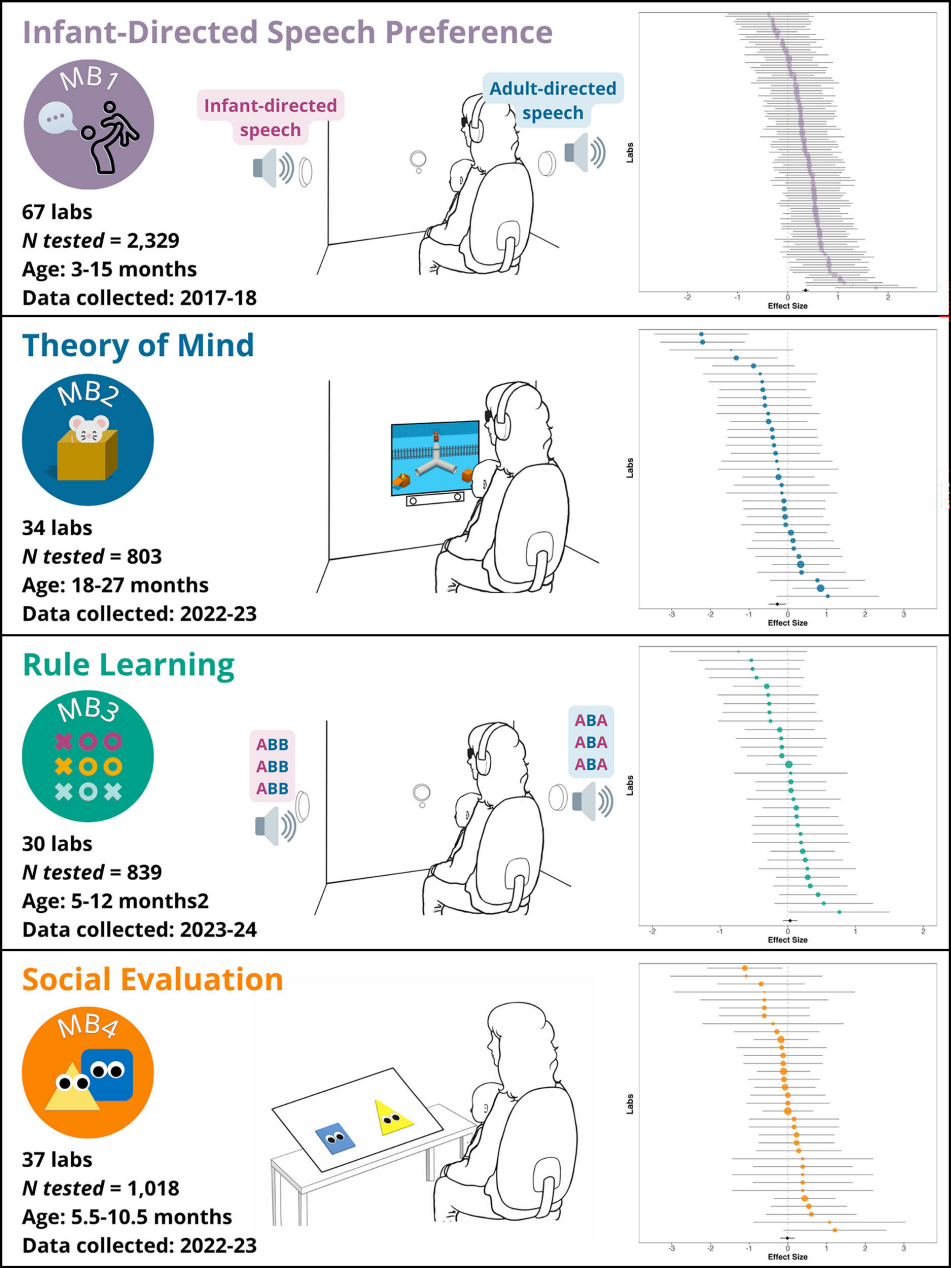
Variability in effect size within and across labs in four ManyBabies studies. This figure illustrates the different methods used in the first four ManyBabies studies (MB1, MB2, MB3, and MB4), including the number of participating labs, sample size, age range of participants, and data collection period. The forest plots show the meta-analytic results for each focal construct. Error bars represent 95% confidence intervals around each lab’s standardized effect size. Method drawings are adapted from Gervain & Werker, 2013^10^, Figure 4, CC-BY-SA-3.0.

In our first collaborative project (MB1: Infant-Directed Speech Preference^3^), we scaled up to 67 labs to investigate what many researchers consider one of the most robust phenomena in developmental science: infants’ preference for infant-directed speech–the high-pitched, melodic way adults in most–but not all–cultures naturally talk to babies. The overall finding was that infants prefer infant-directed speech over adult-directed speech. But responses varied enormously within and across labs: some labs found strong preferences for infant-directed speech; others found weak or negligible effects; a handful found preferences for adult-directed speech; individual responses varied widely (Fig. 1).

Our field has long treated this kind of variability as “noise”, something to be minimized through careful experimental control, larger samples, or better standardization. Sometimes that framing is right as not all variability is theoretically meaningful (e.g., an infant distracted by their sock is not telling us much about speech perception), and some portion of it reflects simple measurement error. But infant behavior is also shaped by factors we typically do not measure, for example, temperament, prior experience, testing context, and caregiver behavior. Whether any given variability is nuisance or signal is an often unanswered empirical question.

ManyBabies addresses this by balancing experimental control with systematic documentation of contextual variation. Across projects, lab protocols instruct sites to standardize core procedural elements while recording a wide range of additional variables drawn from both the empirical literature and the accumulated intuitions of researchers who have spent years conducting infant studies (e.g., testing room features, experimenter characteristics). The scale of ManyBabies, combined with open, de-personalized data sharing, enables pooling individual-level data and systematically testing which factors drive variability. In MB1, for instance, some of the variability we observed related to age, infant language background, and experimental method. Collecting this rich contextual information lets us systematically examine drivers of variability, and in doing so, better understand how development unfolds across individuals and contexts.

## Lesson 2: scaling up *doesn’t* solve all measurement challenges

We initially thought that many of the measurement limitations in infant research–including underpowered designs and idiosyncratic lab effects–might be largely due to small sample sizes, a problem that is solvable by studying more babies across more labs. In practice, larger samples, while solving a critical part of the problem, often brought measurement challenges into sharper focus rather than resolving them. We organize what we have learned around three properties of measurement: the robustness of our effects, the reliability of our measures, and the validity of our interpretations.

### Robustness

The most straightforward benefit of scale is statistical power: larger samples make it possible to detect group-level effects with greater confidence, and to determine whether a finding holds up across different labs and populations. In this sense, ManyBabies has helped. The infant-directed speech preference in MB1, for example, was confirmed across dozens of labs to be a genuinely robust phenomenon. But scale also revealed the fragility of some effects. As shown in Fig. 1, several of our subsequent studies (MB2, MB3, MB4) yielded results that were null or even the opposite of what was predicted. In MB4, for instance, all 37 labs used a single method that had previously demonstrated infants’ preference for prosocial over antisocial agents^4^. However, when results did not support the hypothesis, it was difficult to disentangle whether this reflected the phenomenon we were trying to study, the specific paradigms we chose, or other factors, such as some unspecified impact of the COVID-19 pandemic.

### Reliability

Researchers have long suspected that many infant measures have limited consistency from one measurement to the next, resulting in low test/retest reliability. Larger samples increase statistical power, making it easier to detect effects in groups of infants, but this does not improve the reliability of measurement for any individual infant. A task that is powerful at the group level may tell us little about whether the same infant would respond similarly from one occasion to the next, or whether infants who show stronger responses in one task show stronger responses in another.

This matters because many of the questions we want to ask (e.g., whether early behaviors predict either later development or responses in other tasks) require precise and reliable individual-level measurement. For example, one spin-off study of the MB1 project asked whether infant-directed speech preference in early infancy predicted later vocabulary size^5^. There was no evidence for such a relationship, but this result is difficult to interpret given the low reliability of the preference measure itself^6^. Developing paradigms that yield more consistent individual-level data is a critical next step.

### Validity

The methods we use to study infant cognition often require us to make inferences from behavior. When an infant looks longer at one stimulus than another, what does that actually mean? Surprise? Interest? Confusion? Preference? These interpretations carry different theoretical implications, and our tasks rarely let us distinguish between them. ManyBabies studies are tackling this in two ways. One approach is to improve our fundamental understanding of infants’ behaviors themselves. For example, MB5 investigates the well-documented but poorly understood shift from familiarity to novelty preference in infant looking behavior, attempting to identify factors that explain how a given infant in a given study allocates their attention. A second approach is to triangulate across behavioral, neural, and physiological measures. For example, the ManyBabies 3 rule-learning project tests infants’ sensitivity to abstract patterns using converging behavioral (looking time), neural (fNIRS), and physiological (pupillometry) measures. When different measures converge, our confidence in construct validity grows; when they diverge, we learn something important about what our tasks are actually capturing^7^. Systematically identifying the factors that shape infant behavior and triangulating across multiple measures are particularly critical for a field whose participants cannot report on their own cognition.

In sum, scaling our sample sizes is helpful but insufficient. Resolving measurement challenges might require improving current paradigms or even developing new ones.

## Lesson 3: scaling up can make cultural assumptions visible

Infant development is shaped by cultural context, and multi-site research surfaces this in ways that are easy to overlook in single-lab studies. In planning MB1, we chose to use a single set of North American English stimuli, prioritizing standardization over ecological validity. The data confirmed that this choice carried cultural assumptions: infants who were growing up in North American English contexts showed stronger infant-directed speech preferences than infants learning other languages^3^. Without a basis for comparison, a single-lab finding cannot distinguish universality from a context-specific effect.

The tension between standardization and customization generated spin-off projects designed to disentangle universal from experience-dependent effects, including testing infant-directed speech preference in infants’ native languages, examining how bilingual language exposure shapes the preference, and extending the work to African populations absent from MB1, including an ongoing study by Tsui and colleagues. Even describing participants consistently across cultures required its own dedicated effort: basic demographic categories like socioeconomic status, bilingualism, and race that seem straightforward in a North American context do not translate cleanly across cultures, a challenge addressed in a dedicated methodological paper^8^. We are also broadening the questions we ask, for example, examining early childhood media use across cultural contexts. ManyBabies’ reach into the plurality of human cultural contexts remains limited^9^, but the multi-site structure has made the cultural specificity of our methods visible in a way that single-lab research rarely does.

## Conclusion

ManyBabies began as an effort to establish whether key findings in infant research could be replicated across labs. Over a decade on, that question remains important, but our experience has pushed us toward a broader set of concerns. Scaling up has helped: larger samples and open data sharing have enabled more reliable detection of effects and investigation of sources of variability that single-lab studies cannot. But scale alone cannot resolve the challenges we have encountered. What is also needed is a clearer understanding of how to amplify genuine signals and diminish noise in infant data, how to measure meaningful sources of variability at the individual level, and how to ensure that our questions, stimuli, and procedures are meaningful across the full range of human cultural contexts.

The collaborative structure of ManyBabies has also been essential for generating the data and the cross-lab conversations that made these realizations possible in the first place. Working across hundreds of labs and multiple continents has united researchers around shared problems in a way that single-lab work rarely does. The lessons we have drawn here about variability, measurement, and cultural context are ones that the field is now better positioned to address collectively, and we hope they resonate beyond large-scale collaborations. Single-lab studies also benefit from open data practices, richer documentation of data collection procedures, and collaborative norms that projects like ManyBabies have helped to establish (Table 1).

**Table 1:** Applying open science practices to the three core lessons from ManyBabies studies: Recommendations for future research

Lesson	Open data and materials	Documentation of data collection	Collaborative norms
Lesson 1: variability is data	Share de-identified, individual-level data, standardized metadata, and demographics to enable reanalysis and pooling	Document testing contexts systematically through walk-through videos and structured session logs that capture infant state, caregiver behavior, and room features	Coordinate diverse sampling, preregister shared moderators, and co-design measures
Lesson 2: scaling up doesn’t solve all measurement challenges	Publish stimuli, task code, preprocessing and analysis scripts, and preregistrations for reanalysis and robustness checks	Keep trial-level logs, timestamps, coder notes, calibration/failure reports, and raw recordings when possible. Where feasible, collect repeated measures to assess test-retest reliability	Collaborate to develop new, high-reliability protocols, run cross-site pilots, use shared protocols and centralized quality control, implement inter-rater reliability checks, and triangulate across behavioral, neural, and physiological measures
Lesson 3: scaling up can make cultural assumptions visible	Share stimulus files, translations, and detailed participant descriptors to enable cultural reanalysis	Document culturally relevant participant information (e.g, stimuli familiarity, lab location, language experience) to identify cultural moderators rather than assume universality	Collaborate with local partners at all stages of research, adapt stimuli when appropriate, and ensure equitable credit

Studying so many babies has not always yielded the clear answers we hoped for. Null results, ambiguous findings, and hard questions about how to design our studies and interpret our data have been a recurring part of the ManyBabies experience. But confronting these problems at scale and together has been more productive than addressing them quietly in individual labs. Our field now has a clearer view of where infant behavioral research stands, what current methods can and cannot deliver, and what it will take to make progress. That, too, is something that many babies have taught us.

## Supplementary information

Transparent Peer Review file
